# Peer support for discharge from hospital to community mental healthcare: a cost analysis

**DOI:** 10.1136/gpsych-2024-101671

**Published:** 2025-02-04

**Authors:** Andrew Healey, Akshaykumar Patel, Jacqueline Marks, Stephen Bremner, Rhiannon Foster, Sarah L Gibson, Lucy Pollyanna Goldsmith, Mike Lucock, Julie Repper, Miles Rinaldi, Alan Simpson, Sarah White, Michael Ussher, Steve Gillard

**Affiliations:** 1 Health Services and Population Research, King's College London, London, UK; 2 Wolfson Institute of Population Health, Queen Mary, University of London, London, UK; 3 Kingston Hospital NHS Foundation Trust, Kingston upon Thames, London, UK; 4 Department of Primary Care & Public Health, Brighton and Sussex Medical School, Brighton, UK; 5 School of Health & Psychological Sciences, City, University of London, London, UK; 6 Population Health Research Institute, St George's, University of London, London, UK; 7 Faculty of Health, Science, Social Care and Education, Kingston University, London, UK; 8 School of Human and Health Sciences, University of Huddersfield, Huddersfield, UK; 9 Nottinghamshire Healthcare NHS Foundation Trust, Nottingham, UK; 10 Implementing Recovery through Organisational Change (IMROC), Nottingham, UK; 11 South West London & St George's Mental Health NHS Trust, London, UK; 12 Nordland Hospital Trust, Bodø, Norway; 13 Institute of Psychiatry, Psychology & Neuroscience, King's College London, London, UK; 14 Institute for Social Marketing, University of Stirling, Stirling, UK

**Keywords:** Mental Health Services, Community Mental Health Services

**To the editor:**

Peer workers—people with personal experiences of using mental health services, trained to provide support to others currently using similar services—are increasingly integrated into the workforce of mental health systems internationally.^1^ A meta-analysis of randomised controlled trials of individual peer support in mental health services indicated a modestly significant effect of peer support on measures of self-reported recovery and empowerment compared with care as usual, also showing that peer support was associated with a reduction in the relative risk of psychiatric hospital admission of 14% compared with care as usual.^2^

A report analysing hospitalisation data from six studies internationally suggested that reductions in bed use among people supported by peer workers potentially lead to financial savings in excess of the additional costs of paying peer workers, although sample sizes were small and studies were a mix of randomised, non-randomised and observational designs.^3^ Conversely, an analysis of administrative data in the USA indicated that the total annual Medicaid cost for people using mental health services who were offered peer support was US$6000 higher than for those not offered peer support,^4^ potentially attributed to people with a higher level of need being more likely to be offered peer support or being more able to access other forms of care having first accessed peer support. While there is some evidence of the cost-effectiveness of peer support in other fields—for example, type 2 diabetes care^5^—there remain no formal cost analyses of peer support in mental healthcare. The risk of suicide^6^ and the rates of re-hospitalisation^7^ are at their highest in the 3 months after discharge, suggesting that community-based interventions,^8^ such as peer support, might significantly impact the cost of care at this time.

We undertook a cost analysis of a peer worker intervention aiming to reduce readmission to psychiatric inpatient care after discharge, using data from the largest trial of one-to-one peer support in mental health services to date.^9^

## Methods

We aimed to evaluate whether there was a difference in the total cost of National Health Service (NHS) mental health service contacts over a 12-month period after discharge from inpatient psychiatric care for trial participants who did and did not have access to peer support offered as part of the trial, allowing for the cost of delivering peer support. Secondary analyses examined differences between trial arms in costs relating to subcategories of NHS mental health service utilisation. Adjusting for differential timing of the incidence of costs was not deemed necessary as the estimation of costs did not extend beyond 12 months.

### Research ethics approval

All procedures were approved by the Research Ethics Committee London—London Bridge (London, UK) on 10 May 2016, reference number 16/LO/0470. Written informed consent was obtained from all study participants.

### Setting

The cost analysis was based on a single-blind, randomised controlled trial of peer support for discharge from inpatient to community mental healthcare (ENRICH).^9^ The trial is registered with the ISRCTN registry, ISRCTN10043328.

### Participants

Participants were new admissions to adult acute inpatient wards who had at least one previous psychiatric admission in the preceding 2 years. People were ineligible to participate if they had a diagnosis of any organic mental disorder or a primary diagnosis of eating disorder, learning disability, or drug or alcohol dependency. Participants were randomly assigned to the intervention (peer support plus care as usual) or care as usual in a 1:1 ratio. A full description of the trial design is given in the trial protocol.^10^

### Peer support

Participants allocated to the intervention were offered one-to-one peer support, beginning with at least one session while still an inpatient and continuing with weekly sessions for the first 10 weeks post-discharge, followed by three further fortnightly meetings. Peer workers had received an 8-day manualised training and received weekly group supervision from a peer worker coordinator. Peer support was flexible, comprising a range of individual strengths-based approaches and activities to support connection to community.^9 10^ The intervention was grounded in a set of ‘principles of peer support’ and had been developed using a coproduction approach involving peer workers, people using mental health services and mental health professionals.^11^

### Care as usual

Care as usual was provided by community mental health services, specified as follow-up within 7 days of discharge to determine ongoing care needs.

### Data

#### Service contacts

NHS mental health service contacts were quantified at the individual participant level and were extracted from electronic patient record data supplied by seven participating mental health trusts. Number, type (face-to-face or telephone) and length of contacts with peer workers were collected using an online contact log completed by peer workers after each contact. Details of how service and peer worker contacts were costed are provided in online supplemental table 1.

### Analysis

All analyses of NHS mental health service costs were conducted according to the intention-to-treat principle. We undertook analysis only on participants with observable costs under the assumption that data were missing completely at random.

#### Statistical analysis

Our primary analysis considered the total cost of mental health service contacts as an outcome, defined as the sum of the following subcategories: psychiatric bed day cost; cost of community mental health team contacts; cost of crisis team and emergency department (A&E) psychiatric liaison team contacts; all other service contact costs. We also compared mean costs for each of these cost subcategories separately. A probability distribution with a corresponding mean estimate of the group difference in total cost was obtained using a bootstrapped generalised linear model with baseline covariate adjustment (see online supplemental material for further information).

We tested whether estimated differences in total cost were sensitive to model specifications that allowed for potential non-independence of cost outcome caused by participant clustering around peer support workers in the intervention arm of the trial. A post hoc sensitivity analysis was also carried out to test for the robustness of our main findings to the exclusion of outlying inpatient bed utilisation cost values (defined as costs above the 95th percentile).

## Findings

**Figure 1:**
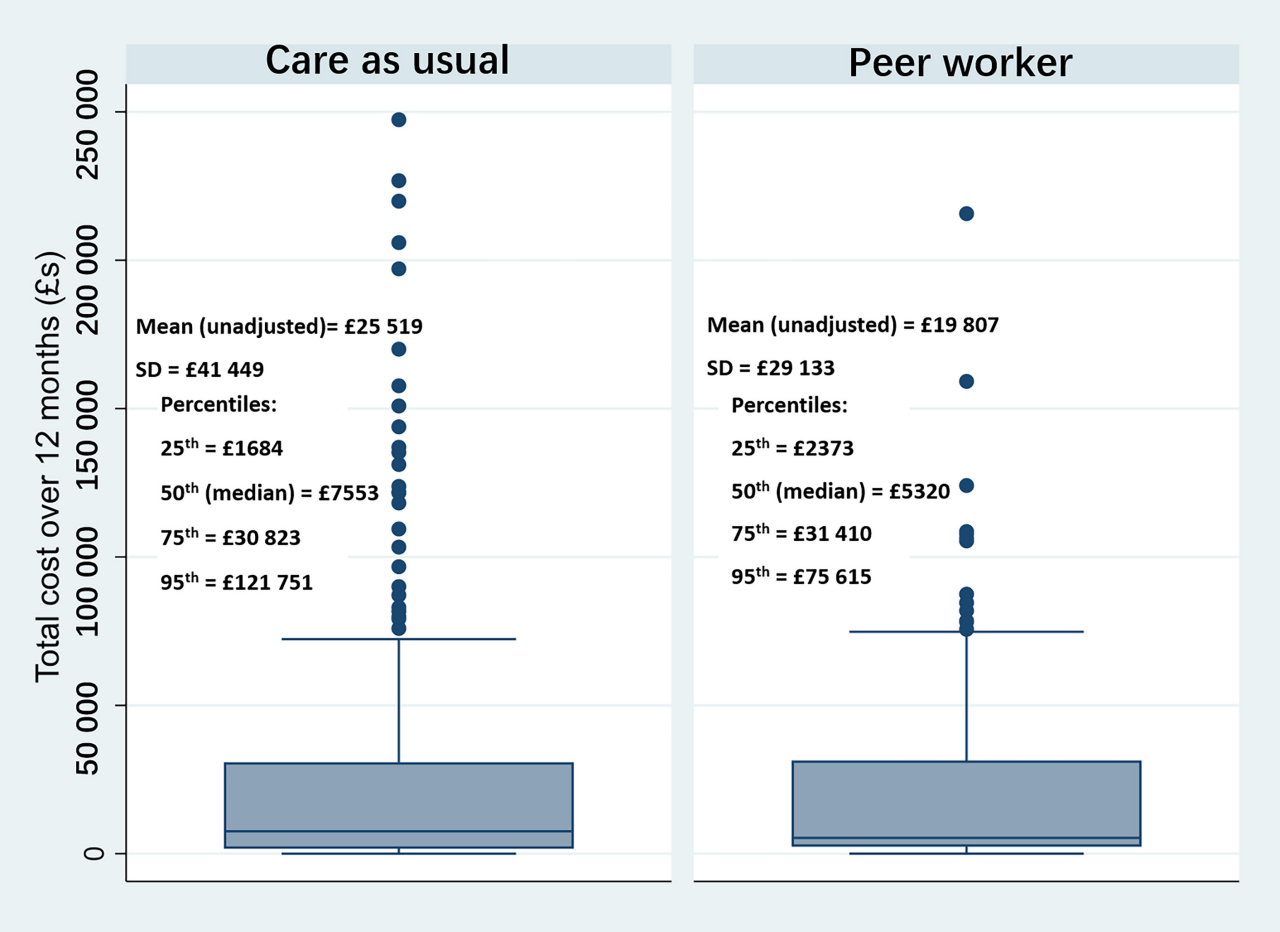
Distribution of total cost by trial group.

Complete mental health service contact data for costing were available for 537 participants (91% of those randomised). See online supplemental figure 1 for a participant flowchart and online supplemental table 2 for descriptive statistics of the cost of NHS mental health service contacts measured over 12 months’ follow-up for peer support and control participants. Figure 1 presents a boxplot of the distribution of total cost by group allocation with additional detail regarding percentile cost values.

Table 1 contains the main results from the analysis carried out on total costs of mental health service contacts over 12 months. Ten further cases were dropped from multivariate cost analyses owing to missing data on baseline covariates. Adjusting for baseline covariates and with reference to mean estimates from bootstrapped statistical models, access to peer support was associated with a 10% reduction in mean total cost of mental health service contacts over 12 months (difference of−£2882 per participant; 95% confidence interval (CI) −£9082 to £3362). There was an 82% probability that access to peer support was associated with lower mean total costs over 12 months given uncertainty due to trial sampling error.

**Table 1:** Adjusted cost comparisons over 12-month follow-up

Variable	Mean cost difference (95% CI)	n
Total cost (including cost of intervention)*	-£2882 (-£9082 to £3362)	527
**Service contactsub-categories†**		
Community mental health team contacts	-£132 (-£380 to £117)	527
Psychiatric bed days	-£3765 (-£9696 to £2167)	527
Crisis team and A&E contacts combined	-£17 (- £209 to £243)	527
All other service contacts	-£65 (-£690 to £560)	527

* Reported statistics based on generalised linear model (gamma error distribution with log link function) estimated on n=5000 bootstrapped samples.

† Reported statistics based on generalised linear model (gamma error distribution with log link function) fitted to trial data. For consistency, each model fitted to the estimation sample used for total costs.

A&Eemergency departmentCIconfidence interval

Secondary analysis of the subcomponents of total cost is also presented in table 1. Adjusting for baseline covariates, the mean cost of psychiatric bed day utilisation was lower for the peer support group (difference of −£3765; 95% CI −£9696 to £2167) with smaller differences detected for community mental health team contacts (−£132; 95% CI −£380 to £117), crisis team and A&E psychiatric liaison team contacts together (−£17; 95% CI −£209 to £243), and for contact with all other service categories (−£65; 95% CI −£690 to £560).

### Sensitivity analysis

Comparing models with and without adjustment for clustering using a multilevel random intercepts model resulted in a small difference in standard error around the estimated group difference in mean total cost and a negligible effect (a 2.5 percent point difference) on the probability of peer support, leading to lower total costs. In post hoc sensitivity analysis, we found that exclusion of the participants in the top 5 per cent of bed day costs resulted in higher mean total costs associated with peer support: a group difference of £608 (95% CI −£3457 to £4579) and a 38% probability that peer support would result in lower mean total costs.

A cost analysis of mental health service contacts over a 12-month period after discharge from inpatient care showed that, on average, and accounting for sampling error in the trial data, the addition of peer support to a participant’s care bundle prior to leaving hospital could reduce the average cost of mental health service contacts by more than £2882 per participant, with 82% probability that peer support was associated with lower total cost of contacts. While group differences in total cost were not statistically significant, as indicated by the associated 95% CI, it is emphasised that the ENRICH trial was not specifically powered to detect statistically significant effects on cost. Cost differences allowed for the additional cost of peer support itself: a mean cost of around £540 per participant over 4 months. Any cost advantage during follow-up was mainly driven by reductions in mean cost of bed day utilisation within the peer support group, supporting and building on findings from earlier research, suggesting that reductions in psychiatric hospital bed use among people supported by peer workers might lead to cost savings that offset additional payments to peer support workers.^3^ Evidence from the main clinical analysis of the ENRICH trial indicated that any cost advantage associated with peer support was not achieved at the expense of inferior clinical outcome; serious adverse event rates (including self-harm) and psychiatric outcomes scores (assessed using the Brief Psychiatric Rating Scale)^12^ were similar for both groups.^9^

Our findings have policy and practice relevance as rates of psychiatric hospital readmission internationally are persistently high,^13^ especially for people with previous admissions (the population in our study),^14^ and inpatient care is expensive. Descriptive results, combined with additional sensitivity analysis, indicate that the lower mean cost for the peer support group was driven by the tail of the distribution; fewer participants with an exceptionally high total cost of service contacts, primarily accounted for by long periods in hospital over follow-up. This may reflect a mediation effect, with peer support preventing readmission, and possibly reducing community mental health service use, for people with more severe symptoms, although exploration of this would require more detailed analysis. Internationally, extended hospital stays—the result of clinical or environmental issues (such as lack of appropriate housing)—remain a problem that is both costly and impactful on experiences of care.^15^ Our findings are indicative of a potential cost benefit of integrating peer support into discharge planning where at least some of these lengthy readmissions are prevented. In addition, secondary analysis from our trial indicated that participants who received more than a minimum amount of the peer support were significantly less likely to be readmitted than a similar group of participants from the control group,^9^ suggesting that peer support might offer greater cost savings than evidenced here if delivery could be optimised.

Our analysis has limitations. A small number of randomised cases (9%) were excluded from the analysis due to missing data on service contact items. Given that our data source was NHS patient record systems rather than questionnaire-based self-report, we think it plausible that missing data would be unrelated to the outcomes of interest in this analysis, and that our group comparisons are robust. We were unable to measure wider service utilisation and costs outside of the NHS mental health system over the 12-month follow-up period so that wider ‘societal’ resource effects of peer support over that period could be evaluated. A full economic evaluation of peer support was also not possible in the absence of participant outcome data at 12 months, including health-related quality of life. An assessment of whether peer support offers a cost-effective alternative to usual care over 12 months when judged against commonly applied economic thresholds for guiding resource allocation in the NHS remains necessary.

We conclude, with some margin of uncertainty, that peer support for discharge from inpatient psychiatric care, offered to people with previous admissions, can lower average costs of mental healthcare use, principally arising from psychiatric hospital bed utilisation, without being harmful to participants.

## supplementary material

10.1136/gpsych-2024-101671 online supplemental file 1

10.1136/gpsych-2024-101671 online supplemental file 2
