# Supplementary figures and images for: Peer support for discharge from hospital to community mental healthcare: a cost analysis
Source: Gen Psychiatr. 2025 Feb 4;38(1):e101671. doi: 10.1136/gpsych-2024-101671 (PMC11795358; doi:10.1136/gpsych-2024-101671)

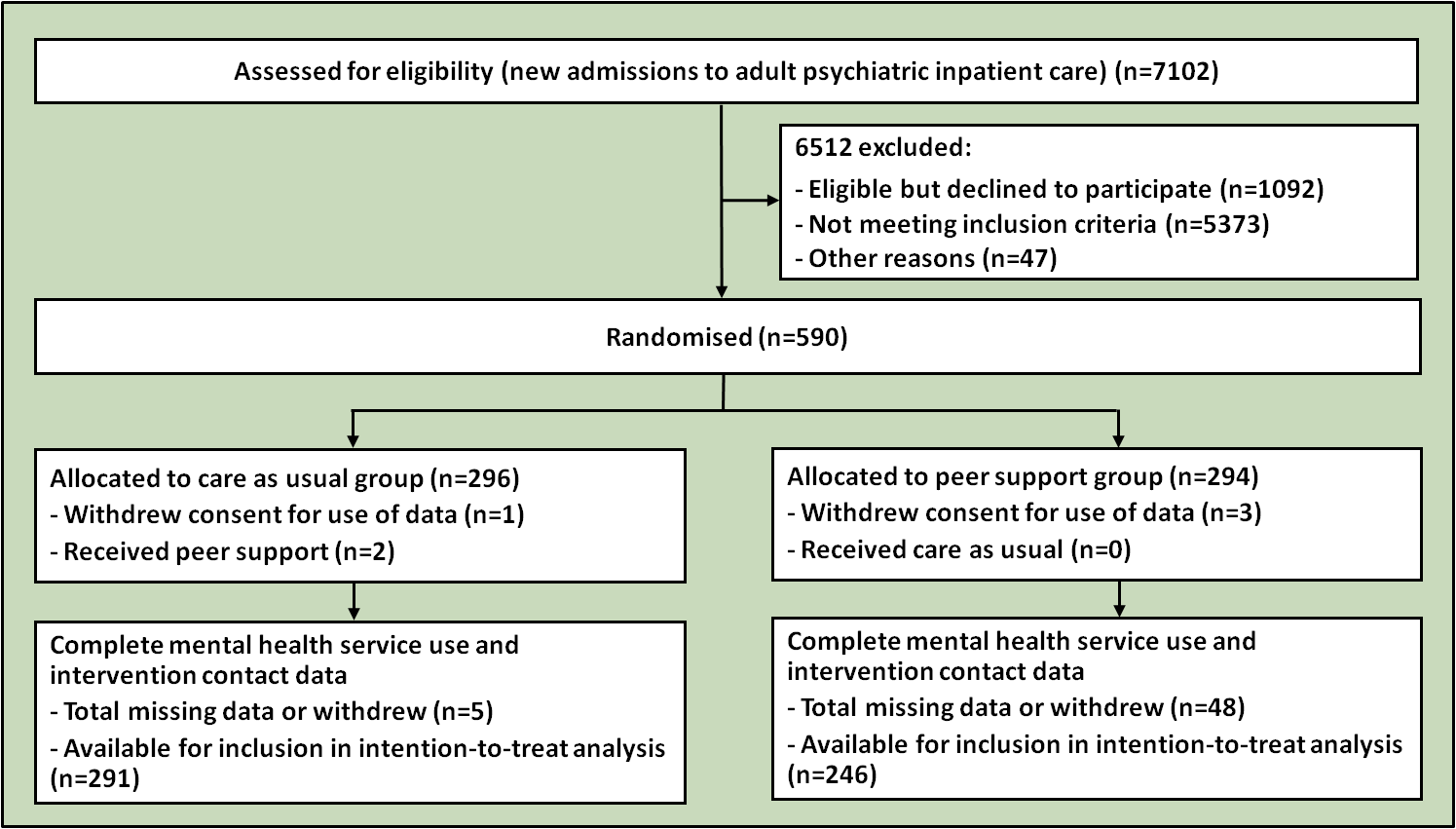

Supplement: online supplemental file 1 [file gpsych-38-1-s001.tif]
