# Supplementary material for: Peer support for discharge from hospital to community mental healthcare: a cost analysis
Source: Gen Psychiatr. 2025 Feb 4;38(1):e101671. doi: 10.1136/gpsych-2024-101671 (PMC11795358; doi:10.1136/gpsych-2024-101671)
Supplement: online supplemental file 2 [file gpsych-38-1-s002.docx]

**Supplementary material**

The manuscript was prepared following the Consolidated Health Economic Evaluation Reporting Standards 2022.[1] A health economic analysis plan was developed and is available on request from the authors. All analyses were carried out using Stata (version 16).[2] The data reported here are available on reasonable request from the authors.

***Unit costs.***

Unit costs required for costing service contacts were obtained from the Unit Costs of Health and Social Care published annually by the Personal Social Services Research Unit and NHS Reference costs (see Supplementary table 1 below).

Activity logs relating to a sample period of 10 working days were completed by peer workers and their supervisors (peer worker coordinators). These were used to identify the ratio between contact and non-contact time allocated to a trial participant, including time spent by peer worker coordinators training and supervising peer workers, and subsequently to estimate a cost per hour of peer support inclusive of costs of training, supervision, and other non-contact time. Peer worker and peer worker coordinator time was costed using the appropriate corresponding NHS salary grading (reported with relevant salary ‘on-costs’ in the Unit Costs of Health and Social Care).(3) Additional overhead costs and capital costs related to employment were applied proportionately in accordance with unit costs reported for similar professionals in the Unit Costs of Health and Social Care.(3) All costs are reported at 2019/20 price levels.

***Supplementary table 1: Unit costs of service contacts (2019-2020 price levels)***

| **Item** | **Cost** | **Unit** | **Source** |
| --- | --- | --- | --- |
| Peer worker | £ 59.77 (including salary and all additional costs of employment). | Hour of face-to-face contact | Estimated from activity log data collected during the study. Annual salary estimated to be  £19 335 at 2019-2020 price levels (excluding on-costs, overheads and capital costs of employment). |
| Peer worker coordinator | £ 54.98 (including salary and all additional costs of employment). | Per hour | Estimated from activity log data collected during the study. Annual salary estimated to be  £39 994 at 2019-2020 price levels (excluding on-costs, overheads and capital costs of employment). |
| Community mental health team | £ 82.00 | Hour of face-to-face contact | 4 |
| Crisis team | £ 85.00 | Hour of face-to-face contact | 4 |
| Psychiatric inpatient admission | £ 430.00 | Bed day | 3 |
| Psychiatric A&E | £ 203.00 | Contact | 3 |
| Community drug and alcohol team | £ 133.00 | Contact | 3 |
| Employment service | £ 33.00 | Contact | 3 |
| Perinatal mental health team | £ 232.00 | Contact | 5 |
| Clinical psychologist (non-consultant) | £ 173.00 | Contact | 5 |
| Art therapy | £ 189.00 | Contact | 6 |
| Psychotherapy | £ 237.00 | Contact | 3 |
| Clinical psychologist | £ 146.00 | Hour of face-to-face contact | 3 |
| Improving access to psychological therapies | £ 96.00 | Contact | 3 |
| Practice nurse | £ 57.00 | Hour of face-to-face contact | 3 |
| Nurse | £ 84.00 | Hour of face-to-face contact | 3 |
| Place of safety | £ 1195.00 | Occurrence | 7 |
| Street triage | £ 60.00 | Contact | 7 |
| Psychiatric nurse | £ 84.00 | Hour of face-to-face contact | 3 |
| Health assistant | £ 45.00 | Hour of face-to-face contact | 3 |
| Social Worker | £ 199.00 | Hour of face-to-face contact | 5 |
| Memory service | £ 445.00 | Hour of face-to-face contact | 3 |
| Criminal justice liaison | £ 255.00 | Contact | 5 |
| Adult forensic service | £ 287.00 | Contact | 5 |
| Eating disorders service | £ 184.00 | Contact | 5 |
| Old age psychiatry team | £ 337.00 | Contact | 5 |
| Child & adolescent mental health services tier 4 | £ 221.00 | Contact | 5 |
| Deaf service for adult with mental health problems | £ 475.00 | Contact | 5 |
| Other specialist mental health services for adults | £ 155.00 | Contact | 5 |
| Psychiatric assessment (non- psychotic severe) | £ 303.00 | Contact | 5 |
| Psychiatric liaison service-adult | £ 183.00 | Contact | 5 |
| Autism service | £ 354.00 | Contact | 5 |
| Occupational therapy team | £ 83.00 | Contact | 5 |
| Housing service | £ 32.00 | Contact | 8 |
| Outpatient attendance – non- - psychiatric* | £ 127.00 | Contact | 5 |
| Impatient admission – non-psychiatric** | £ 1,953.00 | Admission/finished consultant episode | 5 |
| A&E – non-psychiatric | £ 166.00 | Contact | 5 |

| *Weighted average of consultant and non-consultant-led attendances |
| --- |
| **Weighted average of elective, non-elective and short-stay admissions  A&E: Accident and emergency department |

***Statistical analysis – further information***

To estimate differences in mean cost for each trial arm with additional adjustment for variation in baseline covariates we undertook multivariate analysis of total cost (the dependent variable) using generalised linear modelling (GLM) with a logarithmic link function.[9] Total cost was regressed on a dummy variable identifying treatment allocation (1=access to peer support; base category=usual care) and additional baseline covariates: ethnicity; trial site; diagnostic group; total cost of mental health service contacts over 12-months prior to the index admission. Sub-categories of cost (as defined above) were also analysed using GLM methods.

Uncertainty and sensitivity analysis. Non-parametric bootstrap sampling was performed to facilitate quantification of uncertainty around estimated differences in mean total cost arising from trial sampling error. This involved generating n=5000 samples randomly generated from the trial data (with replacement) and subsequently fitting the multivariable model for total cost to each of the samples. This generates a corresponding probability density of estimated difference in mean cost total between the trial arms. The mean value from the distribution is taken to be the ‘best’ estimate (or ‘expected value’) of the covariate adjusted difference. As is conventional with economic analyses of trial data,[10] we use probability values taken from this distribution to characterise uncertainty in conclusions reached regarding differences in total cost that arise from sampling error in the data. We also present 95% confidence limits around the estimated group difference in total cost using the percentile method applied to the bootstrap distribution.[11]

***Supplementary table 2: Cost of NHS mental health service contacts over 12-month follow-up***

This table presents descriptive statistics for the cost of NHS mental health service contacts measured over 12-months’ follow-up for peer support and control participants, including the cost of participant contacts with peer support workers. A positive mean cost of peer worker support is shown for the control arm of the trial on account of two trial participants receiving the intervention in error.

|  | **Peer worker intervention** | | | | **Care as usual** | | | |
| --- | --- | --- | --- | --- | --- | --- | --- | --- |
|  | **Mean** | **Standard dev.** | **Min-max value** | **N** | **Mean** | **Standard dev.** | **Min-max value** | **N** |
| **Peer worker intervention** | £536 | £528 | £0 to £2574 | 251 | £5 | £84 | £0 to £1434 | 296 |
| **Service contacts** |  |  |  |  |  |  |  |  |
| Community mental health teams | £1523 | £1273 | £0 to £8083 | 287 | £1,749 | £1,768 | 0 to £19 883 | 291 |
| Crisis teams | £571 | £1060 | £0 to £8050 | 287 | £554 | £949 | £0 to £6600 | 291 |
| Accident & emergency department (psychiatric-related contacts) | £273 | £709 | £0 to £5278 | 287 | £225 | £569 | £0 to £4669 | 291 |
| Psychiatric bed days | £15 937 | £28 520 | £0 to  £211 560 | 287 | £21 366 | £40 027 | £0 to  £242 529 | 291 |
| Day services | £107 | £758 | £0 to £11 470 | 287 | £324 | £2724 | 0 to £44 020 | 291 |
| Liaison psychiatry | £126 | £408 | £0 to £3660 | 287 | £95 | £367 | £0 to £3294 | 291 |
| Psychological treatment | £401 | £2156 | £0 to £21 279 | 287 | £453 | £2511 | £0 to £36 849 | 291 |
| Psychotherapy | £47 | £472 | £0 to £5925 | 287 | £17 | £278 | £0 to £4740 | 291 |
| Psychiatric assessment | £22 | £129 | £0 to £1212 | 287 | £22 | £229 | £0 to £3636 | 291 |
| Street triage | £3 | £19 | £0 to £180 | 287 | £2 | £13 | £0 to £120 | 291 |
| Criminal justice liaison | £7 | £120 | £0 to £2040 | 287 | £0 | £0 | - | 291 |
| Place of safety (Section 136) | £233 | £913 | £0 to £9560 | 287 | £181 | £1293 | £0 to £20 313 | 291 |
| Forensic psychiatry | £31 | £396 | £0 to £2040 | 287 | £13 | £104 | £0 to £1435 | 291 |
| Community drug and alcohol team | £0 | £0 | - | 287 | £22 | £259 | £0 to £3857 | 291 |
| Occupational therapy service | £101 | £553 | £0 to £7802 | 287 | £161 | £684 | £0 to £6889 | 291 |
| Perinatal mental health | £13 | £173 | £0 to £2784 | 287 | £13 | £172 | £0 to £2784 | 291 |
| All other services | £315 | £1324 | £0 to £13 640 | 287 | £318 | £4182 | £0 to £71 192 | 291 |
| **Total cost over follow-up (including intervention cost)** | £19 807 | £29 133 | £5 to  £215 698 | 246 | £25 519 | £41 449 | £0 to  £247 372 | 291 |

**Source material**

1. Husereau D, Drummond M, Augustovski F, de Bekker-Grob E, Briggs AH, Carswell C, Caulley L, Chaiyakunapruk N, Greenberg D, Loder E, Mauskopf J. Consolidated health economic evaluation reporting standards (CHEERS) 2022 explanation and elaboration: a report of the ISPOR CHEERS II good practices task force. *Value in Health*, 2022; **25**: 10-31.
2. StataCorp. 2019. Stata Statistical Software: Release 16. College Station, TX: StataCorp LLC.
3. Unit costs of Health and Social Care 2019, Personal Social Services Research Unit, University of Kent.
4. Unit costs of Health and Social Care 2010, Personal Social Services Research Unit, University of Kent.
5. NHS Reference Costs 2018/2019. <https://www.england.nhs.uk/national-cost-collection/>
6. NHS Reference Costs 2017/2018. <https://www.england.nhs.uk/national-cost-collection/>
7. Heslin et al (2017) Costs of the police service and mental healthcare pathways experienced by individuals with enduring mental health needs. British Journal of Psychiatry, vol 210, Issue 2.
8. Unit costs of Health and Social Care 2016, Personal Social Services Research Unit, University of Kent.
9. McCullagh P, Nelder J. *Generalised Linear Models*. Chapman and Hall, 1989.
10. Claxton K, Sculpher M, Drummond M. A rational framework for decision making by the National Institute for Clinical Excellence (NICE). *The Lancet*, 2002; **360**(9334): 711-5.
11. Glick HA, Doshi JA, Sonnad SS, Polsky D. *Economic evaluation in clinical trials*. Oxford University Press, 2014.
